# Immunostimulatory Effect of* Zanthoxylum schinifolium*-Based Complex Oil Prepared by Supercritical Fluid Extraction in Splenocytes and Cyclophosphamide-Induced Immunosuppressed Rats

**DOI:** 10.1155/2018/8107326

**Published:** 2018-10-08

**Authors:** Hak Yong Lee, Young Mi Park, Yang Hee Lee, Yang Gyu Kang, Hyang Man Lee, Deuk Seon Park, Hye Jeong Yang, Min Jung Kim, Young-Rae Lee

**Affiliations:** ^1^INVIVO Co. Ltd., 460, Iksandae-ro, Iksan, Jeonbuk 54538, Republic of Korea; ^2^Sandle Co. Ltd., 2000-89 Sincha-ro, Chahwang-myeon, Sancheong, Gyeongnam 52206, Republic of Korea; ^3^Korea Food Research Institute, 245, Nongsaengmyeong-ro, Iseo-myeon, Wanju-gun, Jeollabuk-do 55365, Republic of Korea; ^4^Department of Oral Biochemistry and Institute of Biomaterial-Implant, College of Dentistry, Wonkwang University, 460 Iksandae-ro, Iksan, Jeonbuk 54538, Republic of Korea

## Abstract

Complex oil of* Zanthoxylum schinifolium *and* Perilla frutescens *seed (ZPCO) is used as a traditional medicine due to its pharmacological activities. The aim of this study was to investigate the immunostimulatory effect of ZPCO in isolated splenocytes as well as in an immunosuppressed rat model, which was generated via oral administration of cyclophosphamide. Notably, our results showed that ZPCO exerted an immunity-enhancing effect both* in vitro *and* in vivo*. Specifically, ZPCO treatment enhanced the viability and inflammatory cytokine production of splenocytes and NK cell activity* in vitro. *Moreover, this product improved host defense under immunosuppressive conditions by increasing the number of immune cells and promoting the expression of cytokines involved in immune responses. Our results suggest that complex oil including* Z. schinifolium *should be explored as a novel immunostimulatory agent that could potentially be used for therapeutic purposes or as an ingredient in functional foods.

## 1. Introduction

*Zanthoxylum schinifolium *is an aromatic plant that is widely used as a spice for cooking. Notably, the extract of this plant is also used as a crude drug for treating pain in East Asian countries [1]. Recently, several phytochemical studies conducted on* Z. schinifolium *focused on the essential oils, coumarins, flavonoids, and alkaloids in the fruits and leaves of the plant [2]. Furthermore,* Z. schinifolium* extracts were shown to exert various pharmacological effects, including antiplatelet aggregation [3], antioxidant [4], and antitumor activities [5], and were found to inhibit the production of monoamine oxidase [6]. Although a recent report showed that an ethanol extract of* Z. schinifolium* exerted an anti-inflammatory effect on human cells [7], there have yet to be any reports on the effects of Complex oil including* Z. schinifolium* on the immune response.

The immune system comprises various organs such as the thymus, bone marrow, lymph nodes, and spleen. The body becomes prone to infections caused by invading organisms when the immune system is compromised. Thus, it is critical to identify treatments for enhancing the immune response of immunocompromised individuals to protect against infections by pathogenic organisms. In a recent study, natural plants and their immunomodulatory components were studied in an effort to discover potential immunoenhancing agents for use as ingredients in functional foods [8]

Cancer is one of the leading causes of death worldwide. Many drugs used for cancer treatment are associated with harmful side effects such as damage to the immune system [9]. Furthermore, chemotherapeutic strategies for cancer treatment are associated with high systemic toxicity and drug resistance, which limit treatment success in most cases [10]. Accordingly, new therapeutic strategies have been developed that do not damage the immune system of patients undergoing cancer treatment [11, 12], and efforts are being made to research natural compounds that can improve immunity [13]. The role of activated immune cells in the defense against cancer cells has been extensively studied. Moreover, evidence that activated immune cells recognize and eliminate cancer cells, including cancer cells resistant to drugs used for chemotherapy, has been reported [14, 15].

Cyclophosphamide (Cy) is a commonly used antineoplastic agent which often results in immunosuppression, myelosuppression, and cytotoxic effects [16–18]. Administration of Cy can lead to a sudden change in Th1/Th2 bias, resulting in immunosuppression [19]. In previous studies, the immunological effects of Cy were found to decrease the absolute number of T cells, proliferation of lymphocytes, Th1 cell produced cytokines (IL-2, IL-12, and IFN-*γ*), and Th2 produced cytokine (IL-4) [20].

In recent studies, much effort has been directed to discover novel natural compounds with immune-enhancing effects. The role of activated immune cells in the defense against cancer cells has been extensively studied. Moreover, it has been reported that activated immune cells recognize and eliminate cancer cells, including drug resistant cancer cells [14, 15]. In the present study, we investigated the* in vitro* and* in vivo* immunostimulatory effects of ZPCO in isolated splenocytes and immunosuppressed rats.

## 2. Materials and Methods

### 2.1. Preparation of ZPCO

ZPCO was manufactured by Sandle Co. Ltd. (Sancheong-Gun, Gyeongnam, Korea). The oil was prepared as follows. Briefly, the aerial parts of* Z. schinifolium *and* Perilla frutescens *seed were collected from Hansalim Contract Farming complex in Sancheong, Gyeongnam, Republic of Korea, dried, and powdered. The powder of* Z. schinifolium *(150 kg) and* Perilla frutescens* seed (150 kg were extracted with a supercritical CO2 extractor [21], and of extracted oil of 75 L was obtained. The sample was incubated at 50°C and 250 bars for 10 min. Extraction was then performed by passing CO_2_ (99.9%) through the column at a flow rate of 2.0 ml/min to obtain the oil.

### 2.2. Sample Preparation

Fat extract: the homogenized specimen was weighed and placed in a Mojonnier tube. About 100 mg of pyrogallol and 2 ml of internal standard solution were added to the tube. Subsequently, boiling water, 2 ml of ethanol, and 10 ml of 8.3 M hydrochloric acid solution were added and mixed well; the tube was sealed and immersed in a water bath at 70–80°C for 40 min. Preparation of test solution: the fat, extracted with 2–3 ml chloroform and 2–3 ml diethyl ether, was dissolved and transferred to a 15-ml test tube. The solution was concentrated with nitrogen at 40°C, and 2.0 ml of 7% trifluoroborane methanol solution and 1.0 ml of toluene were added. The mixture was heated in a 100°C oven for 45 min and then cooled. Subsequently, 5.0 ml of distilled water, 1.0 ml of hexane, and about 1.0 g of anhydrous sodium sulfate were added, and the mixture was allowed to stand with shaking. The separated supernatant was dehydrated to prepare a test solution.

### 2.3. GC Analysis

ZPCO analysis was performed using a gas chromatograph (GC 7890B, Agilent Technology, USA) equipped with a flame ionization detector (FID). SP-2560 (100 m (100 m × 0.25 mm × 0.2 m) was used for column separation; the column flow was 0.75 ml/min and the split ratio was 200:1. The temperatures of the insert and the detector were 225°C and 285°C, respectively. The temperature of the oven was maintained at 100°C for 4 min, subsequently elevated up to 240°C, at the rate of 3°C/min, and maintained at this temperature for 15 min.

### 2.4. Animals

Five-week-old male Sprague Dawley (SD) (n = 36) rats were purchased from Samtaco Inc. (Osan, Gyeonggi-do, Korea) and adapted to the following conditions for 7 days: 12-h light/12-h dark cycle; temperature, 23 ± 1°C; humidity, 50 ± 5%; and illumination, 150–300 lux. The animals were allowed* ad libitum* access to food (Purina diet; Purina Korea, Seongnam, Gyeonggi-do, Korea) and water. Splenocytes were collected from one rat for the study. The remaining 35 rats were then randomly assigned to five groups (7 rats per group). The protocols used for these animal studies were approved by the Committee on Care and Use of Laboratory Animals of Wonkwang University (Iksan, Jeollabuk-do, Korea; approval no. WKU16-19).

### 2.5. Cell Culture

The spleen of an 8-week-old SD rat was aseptically dissected to obtain splenocytes. The spleen was gently pressed with forceps and then forced through a 70-*μ*m cell strainer (SPL Life Sciences, Pocheon-si, Gyeonggi-do, Korea). The cells were collected and washed three times in Roswell Park Memorial Institute (RPMI)-1640 (Invitrogen, Carlsbad, CA, USA) by centrifugation (80 ×* g*, 3 min, 4°C). Next, the cells were treated with red blood cell lysis buffer (Sigma-Aldrich, St. Louis, MO, USA). Isolated splenocytes were maintained in RPMI-1640 containing 10% fetal bovine serum (FBS) and 1% antibiotics (penicillin, streptomycin) (Invitrogen) in a 5% CO_2_ incubator.

### 2.6. Cell Viability

Cell viability assays were performed using a WST-1 Assay Kit (ITSBio, Seoul, Gyeonggi-do, Korea), according to the manufacturer's instructions. Briefly, splenocytes (2 × 10^5^ cells/well) were seeded into 96-well plates and incubated at 37°C for 4 h to allow for cell stabilization. Next, the cells were treated with ZPCO (0, 5, 10, 30, 50, 100, or 300 *μ*g/ml) and cyclophosphamide (CY; 1600 *μ*g/ml) or lipopolysaccharide (LPS; 10 *μ*g/ml) and incubated for 24 h in a 5% CO_2_ incubator. Each experiment was performed in triplicate. Splenocyte viability rate was assessed using a WST-1 Assay Kit and a Sunrise™ enzyme-linked immunosorbent assay (ELISA) plate reader (Tecan, Männedorf, Switzerland).

### 2.7. NK Cell Activity Assay

YAC-1 cells obtained from the American Type Culture Collection (ATCC, Manassas, USA) were used as target cells for NK cell activity assay, and splenocytes were isolated from control or ZPCO-treated groups for use as effector cells. Splenocytes were cocultured with YAC-1 cells in 96-well plates at a ratio of effector cells to target cells (10:1, 20:1, and 50:1) and cultured in a 5% CO_2_ incubator at 37°C for 24 h. YAC-1 viability rate was assessed using a WST-1 Assay Kit and a Sunrise™ enzyme-linked immunosorbent assay (ELISA) plate reader (Tecan, Männedorf, Switzerland). The NK cell activity was calculated as the survival rate of YAC-1 compared to that of the control group.

### 2.8. Cytokine Levels in Splenocytes

Splenocytes (2 × 10^5^ cells/well) were seeded into 96-well plates with RPMI-1640 containing 10% FBS and 1% antibiotics (growth media), after which ZPCO (0, 1, 10, 100, or 250 *μ*g/ml) and LPS (10 *μ*g/ml) were added to the wells. The cells were then incubated for 24 h in a 5% CO_2_ incubator. Each experiment was performed in triplicate. The levels of tumor necrosis factor- (TNF-) *α*, interferon- (IFN-) *γ*, interleukin- (IL-) 2, and IL-12 in the culture medium from each well were then measured using Cytokine Activation Analysis Kits (R&D Systems, Minneapolis, MN, USA), according to the manufacturer's instructions. The results were measured using an ELISA reader.

### 2.9. Determination of Nitric Oxide (NO) Production

After various treatments, NO in the culture supernatants was measured with the addition of 100 *μ*l of Griess reagent (1% sulfanilamide and 0.1% N-[1-naphthyl]-ethylenediamine dihydrochloride in 5% phosphoric acid) to 100 *μ*l of each sample. The NO concentration was determined at 540 nm using NaNO2 as a standard.

### 2.10. Complete Blood Count (CBC) and Cytokine Analyses

SD rats were orally administered ZPCO (0, 30, 100, or 300 mg/kg/day) and CY (5 mg/kg, once per day) for 28 days. Meanwhile, rats administered saline were used as a control group. After the final administration of the various drugs, the rats were weighed and anesthetized via intraperitoneal injection of 2,2,2-tribromoethanol (Sigma-Aldrich). Whole blood was collected through the abdominal vena cava into ethylenediaminetetraacetic acid (EDTA) microtubes. Next, the rats were sacrificed by brief exposure to 100% CO_2_, followed by cervical dislocation. The numbers of white blood cells (WBCs), lymphocytes, and neutrophils in each whole blood sample were measured using a Hemavet 950 system (Drew Scientific Group, Dallas, TX, USA). In addition, midrange absolute counts (MID), which generally includes monocytes, eosinophils, and basophils, were determined. The plasma levels of TNF-*α*, IFN-*γ*, IL-2, and IL-12 were quantified using ELISA kits (R&D Systems), according to the manufacturer's instructions.

### 2.11. Histological Analysis

After the animals were sacrificed, their organs (liver, kidney, thymus, and spleen) were removed, weighed, and fixed in 10% neutral buffered formalin. The organs were then processed for embedding in paraffin, after which they were sectioned into 4–7-*μ*m-thick slices using a microtome (Thermo Scientific, Waltham, MA, USA). The sectioned tissues were then stained with hematoxylin and eosin. Tissue damage was assessed under an optical microscope (Olympus, Fukuoka, Japan).

### 2.12. Statistical Analysis

Results were analyzed by one-way analysis of variance (ANOVA) and Duncan's multiple range tests using SAS software (version 9.3; SAS Institute Inc., Cary, NC, USA). P values < 0.05 were considered statistically significant.

## 3. Results and Discussion

### 3.1. HPLC Analysis of ZPCO

**Figure 1:**
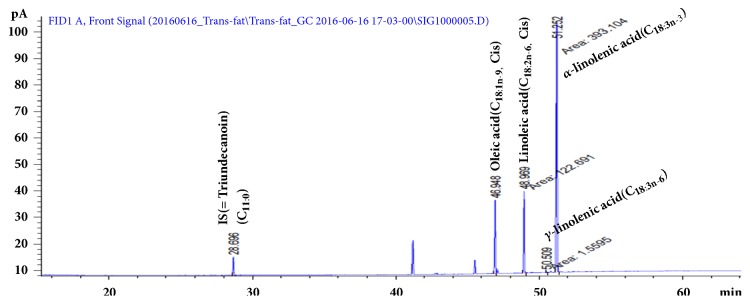
Gas chromatography profile of ZPCO. Large amounts of oleic acid, linoleic acid, and linolenic acid were detected in ZPCO.

The extract was subjected to GC profiling to analyze the constituents of the* ZPCO*. A composition analysis of the* ZPCO* revealed that palmitic acid (19%), oleic acid (35%), linoleic acid (23%), and linolenic acid (16%) constituted more than 90% of fatty acid content [22]. The ZPCO extract used in this study also contains a large amount of oleic acid, linoleic acid, and linolenic acid (Figure 1). Fatty acids are implicated in pathologies, including cardiovascular disease, metabolic diseases such as cancer, diabetes, and inflammatory diseases [23, 24].

### 3.2. ZPCO Stimulates Splenocyte Viability

**Figure 2:**
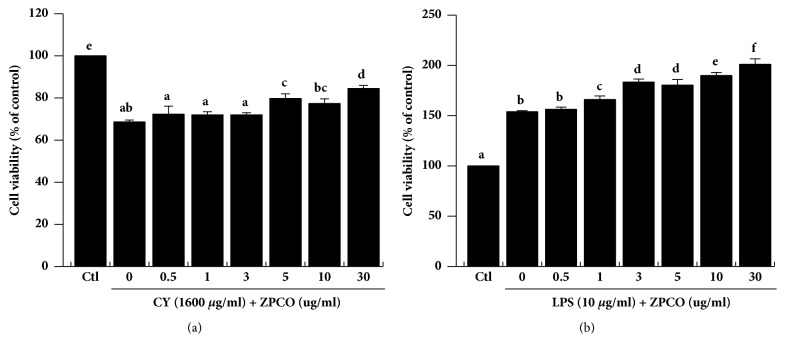
Effect of ZPCO on splenocyte viability. Cells were seeded into 96-well plates, followed by treatment with ZPCO (0, 5, 10, 30, 50, 100, or 300 *μ*g/ml) and cyclophosphamide (CY; 1600 *μ*g/ml) or lipopolysaccharide (LPS; 10 *μ*g/ml). Next, the cells were incubated for 24 h in a 5% CO_2_ incubator, after which viability was assessed. Bars labeled with different superscripts have significantly different values (P < 0.05). Data are presented as means ± standard errors (n = 3).

Proliferation of lymphocyte and macrophage is an early event in the activation of cellular and humoral immune responses. Splenocytes are composed of various cell types, including macrophages, dendritic cells, and T and B lymphocytes, which have different immune functions [15]. Generally, LPS causes activation of polyclonal B cells and stimulation of monocyte, dendritic cell, and macrophages. Furthermore, it has been reported that LPS activates T cells [25–27]. In addition, CD4 T cells have been shown to express toll-like receptor-4 (TLR4), a receptor for LPS [28, 29]. Stimulation of splenocyte viability ultimately increases the release of cytokines, potentially explaining the immune enhancement and antitumor activity [30, 31]. Therefore, in our study, splenocytes were incubated with CY (1600 *μ*g/ml) or LPS ((10 *μ*g/ml), and ZPCO (0, 5, 10, 30, 50, 100, or 300 *μ*g/ml) for 24 h, after which cell viability was measured. This was done to assess levels of splenocyte proliferation following each treatment. Notably, ZPCO treatment increased splenocyte viability in a dose-dependent manner (Figure 2). In addition, ZPCO treatment significantly attenuated the reduction in cell viability observed in splenocytes treated with CY and LPS but, ZPCO alone-treated group did not any significant change in viability (Supplementary Figure 1).

### 3.3. Evaluation of the Effect of ZPCO on NK Cell Activity Assay

**Figure 3:**
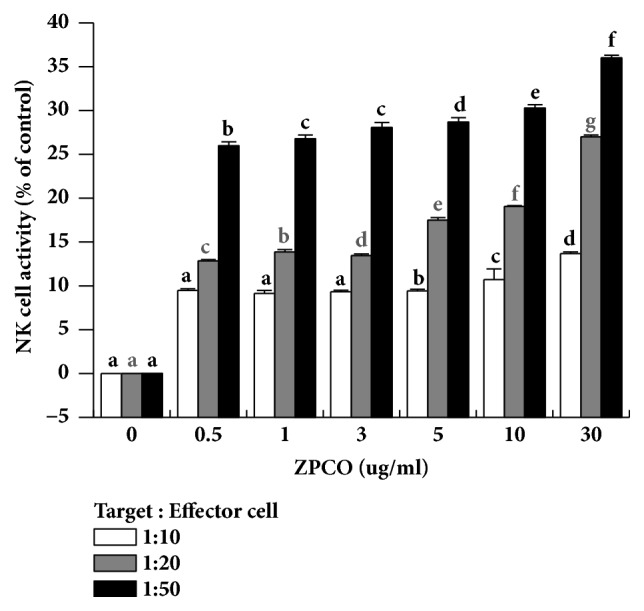
Effect of ZPCO on NK cell activity. Splenocytes were cocultured with target cells (YAC-1) in 96-well plates, followed by treatment with ZPCO (0, 5, 10, 30, 50, 100, or 300 *μ*g/ml) and incubated for 24 h in a 5% CO_2_ incubator with a ratio of effector to target cells of 10:1, 20:1, and 50:1. The NK cell activity was calculated as the survival rate of YAC-1 compared to that of the control group. Bars labeled with different superscripts have significantly different values (P < 0.05* versus *control). Data are presented as means ± standard errors (n = 3).

NK cells are activated by stimulation of cytokines and chemokines and have been shown to play a central role in the regulation of tumor growth and metastasis [32]. NK cells are a major population of cytotoxic lymphocytes and play an important role in the defense against cancer and viruses [33, 34]. NK cell activity assays have been used to assess the effects of functional foods on nonspecific cell-mediated immunity. Therefore, we confirmed the effects on NK cell activity by ZPCO. Splenocyte cytotoxicity was tested against NK-sensitive tumor cells (Yac-1). As shown in Figure 3, the NK cell activity was significantly increased after exposure to the ZPCO. The results indicated that the ZPCO can regulate the innate immune response against tumor cell.

### 3.4. ZPCO Increases LPS-Induced Cytokine Expression in Splenocytes

**Figure 4:**
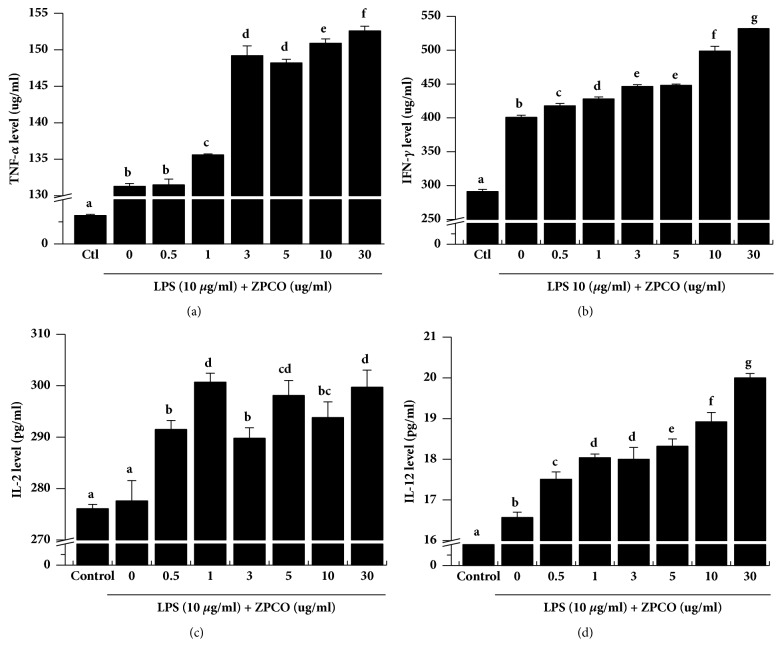
Effect of ZPCO on the concentration of cytokines in splenocytes. Cells were seeded into 96-well plates, followed by treatment with ZPCO (0, 5, 10, 30, 50, 100, or 300 *μ*g/ml) or lipopolysaccharide (LPS; 10 *μ*g/ml). Next, the cells were incubated for 24 h in a 5% CO_2_ incubator, after which the levels of TNF-*α*, IFN-*γ*, IL-2, and IL-12 secretion into the culture medium were analyzed. Bars labeled with different superscripts have significantly different values (P < 0.05* versus *control). Data are presented as means ± standard errors (n = 3).

Cytokines, which play important roles in immune modulation, inflammation regulation, lymphocyte differentiation, host defense against bacterial infection, cell survival, cell death, and immune responses [35–37], are released by several immune cell types. LPS stimulates CD4+T cells and d, APCs such as monocyte, macrophages, and dendritic cells, which secrete a variety of cytokines such as IFN-1, TNF-*α*, and IL-12 [25, 27, 38]. Furthermore, IL-12 plays an essential role in the differentiation of naive T cells into Th1 cells and activation of natural killer cells [39]. In the present study, we evaluated the* in vitro *effect of ZPCO on the production of cytokines associated with immune responses by incubating splenocytes with ZPCO (0, 5, 10, 30, 50, 100, or 300 *μ*g/ml) and LPS (10 *μ*g/ml). As shown in Figure 4, ZPCO-treated splenocytes produced higher levels of TNF-*α*, IFN-*γ*, IL-2, and IL-12 than did the non-ZPCO-treated and control splenocyte populations. ZPCO alone-treated splenocytes slightly increased production of TNF-a, IFN-y, IL-2, and IL-12 (Supplementary figure 2).

### 3.5. Effect of ZPCO on NO Production in Splenocytes

Splenocyte activation can play a role in novel immunotherapeutic approaches for the treatment of cancer [15]. NO regulates host immunity as a modulator of T-lymphocyte responses. There are some controversial reports about the immunomodulation of NO. Badovinac et al. (2000) found that macrophage-derived NO had an effect in promoting IL-2-induced splenocyte growth at low concentrations [40]. Therefore, we examined whether ZPCO was able to stimulate the growth and functional activation of splenocytes. We confirmed that NO reduced by CY is restored by ZPCO (Supplementary figure 3). This result suggests that ZPCO could activate the splenocyte-mediated immune response and growth.

### 3.6. ZPCO Treatment Results in Increased Numbers of Immune Cells in Rats

**Figure 5:**
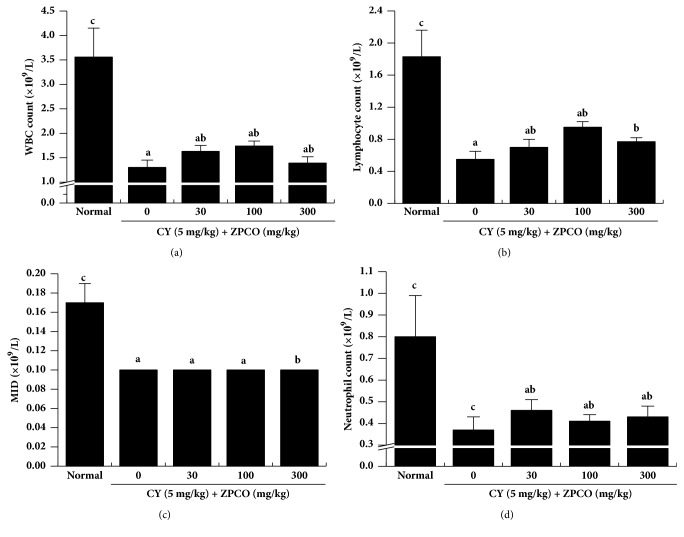
Effects of ZPCO on white blood cell (WBC), neutrophil, and lymphocyte counts and on midrange absolute counts (MID) in the whole blood of Sprague Dawley (SD) rats. SD rats were treated with saline, cyclophosphamide (CY; 5 mg/kg/day), and oral ZPCO (0, 30, 100, or 300 mg/kg/day) once daily for 28 days, after which whole blood samples were collected for analysis. The levels of WBCs, neutrophils, and lymphocytes in the blood samples were determined using a Hemavet 950 system. MID was also measured. Bars labeled with different superscripts have significantly different values (P < 0.05* versus* control. Data are presented as means ± standard errors (n = 7).

Immunosuppression is an indication that the defense system is not strong enough to defend a host against various infections. As people age, their immune system becomes weaker. For instance, immune tissues shrink, and the number of white blood cells decreases [41]. Immune responses are elicited by immune cells such as T and B lymphocytes, monocytes, and macrophages. Therefore, these cells play a critical role in immune modulation [35–37]. In the present study, we found that ZPCO treatment resulted in increased numbers of WBCs, lymphocytes, and neutrophils in immunosuppressed rats (Figure 5), which we believe was due to the immunostimulatory effect of ZPCO.

### 3.7. ZPCO Treatment Results in Increased Plasma Levels of Immune-Related Cytokines in Immunosuppressed Rats

As mentioned earlier, cytokines are crucial in the regulation of inflammatory responses. In particular, TNF-*α*, which is produced by T and B lymphocytes, natural killer cells, and macrophages, modulates inflammation and host defense by inhibiting bacterial infection [42] and suppressing acute stress [41]. INF-*γ* and IL-2 are naturally produced by T-helper cells [43]. While INF-*γ* is a natural immune mediator that activates monocytes/macrophages and promotes the expression of major histocompatibility complex molecules [44], IL-2 enhances T-lymphocyte responses by regulating homeostasis and the differentiation of immature T lymphocytes into regulatory T lymphocytes [45]. Lastly, IL-12 is produced by various immune cells, including dendritic cells, macrophages, neutrophils, and human B-lymphoblastoid cells [46] and has been shown to induce the differentiation of naive T lymphocytes into Th1 lymphocytes and to promote the production of TNF-*α* and IFN-*γ* from T lymphocytes and natural killer cells [47].

**Figure 6:**
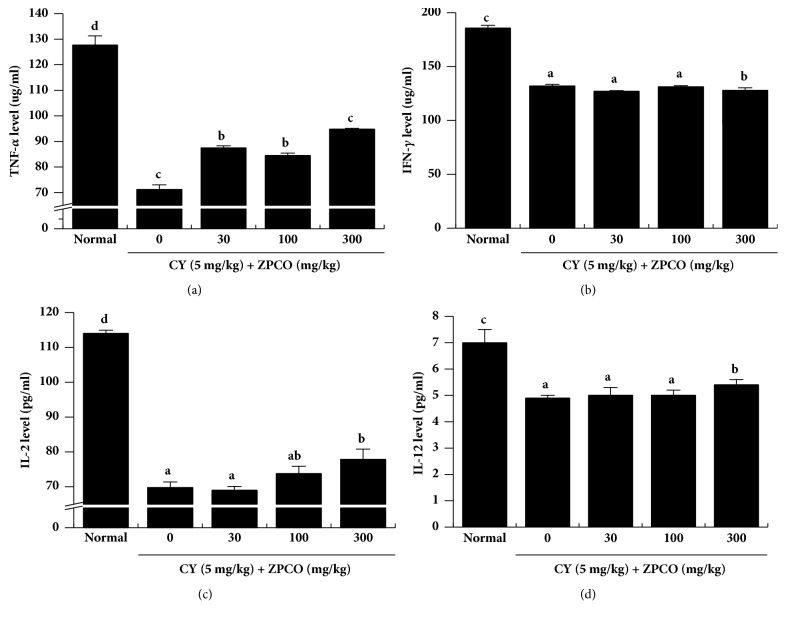
Effect of ZPCO on the plasma levels of immune-related cytokines in the serum of Sprague Dawley (SD) rats. SD rats were treated with saline, cyclophosphamide (CY; 5 mg/kg/day), and oral ZPCO (0, 30, 100, or 300 mg/kg/day) once daily for 28 days, after which serum levels of TNF-*α*, IFN-*γ*, IL-2, and IL-12 were quantified using ELISA kits. Bars labeled with different superscripts have significantly different values (P < 0.05* versus* control). Data are presented as means ± standard errors (n = 7).

Studies are being actively conducted to identify immunomodulators in natural products [48–51]. In the present study, to evaluate the effects of ZPCO on the expression of immune-related cytokines, we measured the concentrations of TNF-*α*, IFN-*γ*, IL-2, and IL-12 in the plasma rats treated with ZPCO and/or Cy (Figure 6). The plasma levels of each cytokine were lower in CY-treated animals than in the normal rats (saline-treated). In addition, among the treated animals, the ZPCO-treated groups typically exhibited significantly higher plasma levels of TNF-*α* and IL-2 than the non-ZPCO-treated groups. Furthermore, the group treated with 300 mg/kg ZPCO showed a slight increase in IL-12 levels.

### 3.8. ZPCO Protects Spleen Damage in CY-Induced Immunosuppressed Rats

**Figure 7:**
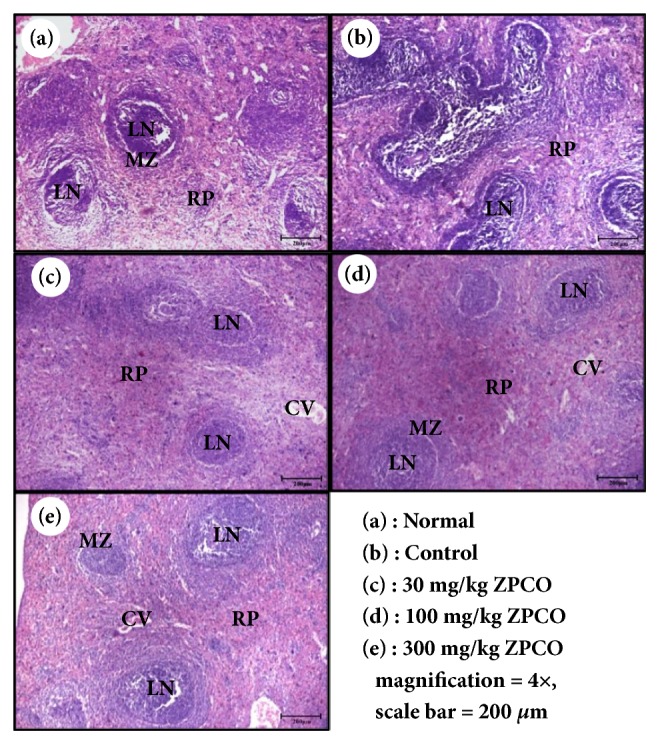
Effect of ZPCO on immunity-associated spleen damage in Sprague Dawley (SD) rats. SD rats were oral administrated with saline, cyclophosphamide (CY; 5 mg/kg/day), and ZPCO (0, 30, 100, or 300 mg/kg/day) once daily for 28 days, after which spleen damage was analyzed histologically. Representative images of the sectioned spleens of (a) normal rats (saline treatment), (b) control rats (treated with only CY), and (c–e) ZPCO-treated rats [treated with CY and (c) 30 mg/kg, (d) 100 mg/kg, or (e) 300 mg/kg ZPCO]. Scale bar = 200 *μ*m. CV, central vein; LN, lymph nodule; MZ, marginal zone; RP, red pulp.

Figure 7 shows the results obtained via histological analysis of rat spleens. We found that the tissues from the control group showed white pulp surrounding the central vein. Additionally, a marginal zone was observed between the white and red pulps (Figure 7(a)). However, tissues from the CY-treated mice showed an obscured marginal zone. In addition, irregular cell condensates were observed in the red pulp area (Figure 7(b)). For rats that were treated with ZPCO, especially at a high concentration, a clear marginal zone between the white and red pulps was present, whereas cell condensates were hardly observed (Figure 7(e)). Together, these results indicate that ZPCO stimulated innate and adaptive immunity by facilitating the production of immunity-related cytokines and improving the histopathological characteristics of spleens that had undergone CY-induced damage.

## 4. Conclusions

The results of the* in vitro* and* in vivo* studies presented here suggest that ZPCO stimulates the production of certain innate immune cell types as well as specific cytokines and NK cell activity that together improve immunity and host defense under immunosuppressive conditions. These findings indicate that* Z. schinifolium *should be explored as a potential novel immunostimulatory agent for use in functional foods and medicines.

## Data Availability

The data used to support the findings of this study are included within the article.

## Supplementary Materials

Supplementary Materials Supplementary Figure 1: effect of ZPCO on splenocyte viability. Cells were seeded into 96-well plates, followed by treatment with ZPCO (0, 5, 10, 30, 50, 100, or 300 *μ*g/ml). Next, the cells were incubated for 24 h in a 5% CO2 incubator, after which viability was assessed. Bars labeled with different superscripts have significantly different values (P < 0.05). Data are presented as means ± standard errors (n = 3). Supplementary Figure 2: effect of ZPCO on the concentration of cytokines in splenocytes. Cells were seeded into 96-well plates, followed by treatment with ZPCO (0, 5, 10, 30, 50, 100, or 300 *μ*g/ml). Next, the cells were incubated for 24 h in a 5% CO2 incubator, after which the levels of TNF-*α*, IFN-*γ*, IL-2, and IL-12 secretion into the culture medium were analyzed. Bars labeled with different superscripts have significantly different values (P < 0.05 versus control). Data are presented as means ± standard errors (n = 3). Supplementary Figure 3: effects of ZPCO on NO production in splenocytes. Splenocytes were incubated with ZPCO (0, 10, 30, 50, 100, and 300 ug/ml) or cyclophosphamide (1600 *μ*g/ml) for 24 h, and NO concentrations in the culture supernatants were assessed using the Griess assay. Bars labeled with different superscripts have significantly different values (P < 0.05). Data are presented as means ± standard errors (n = 3).
