# Supplementary figures and images for: Immunostimulatory Effect of Zanthoxylum schinifolium-Based Complex Oil Prepared by Supercritical Fluid Extraction in Splenocytes and Cyclophosphamide-Induced Immunosuppressed Rats
Source: Evid Based Complement Alternat Med. 2018 Oct 8;2018:8107326. doi: 10.1155/2018/8107326 (PMC6196913; doi:10.1155/2018/8107326)

Supplementary Figure 1

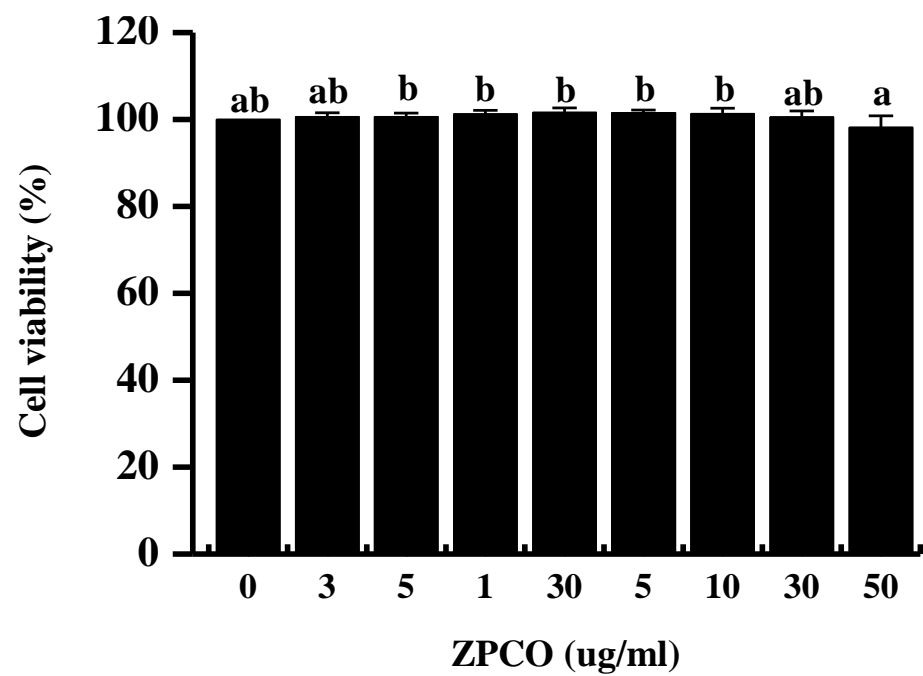

Supplementary Figure 2

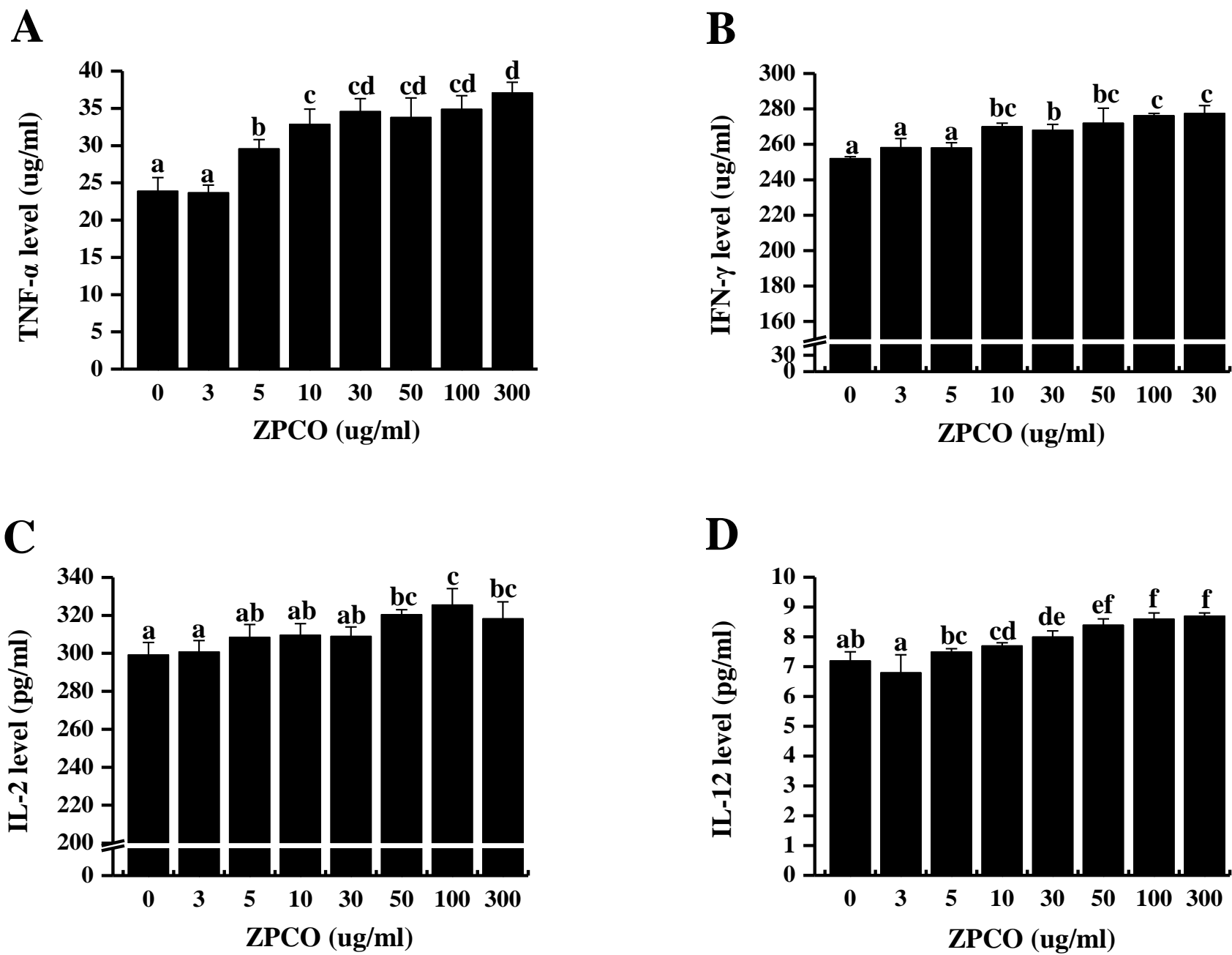

Supplementary Figure 3

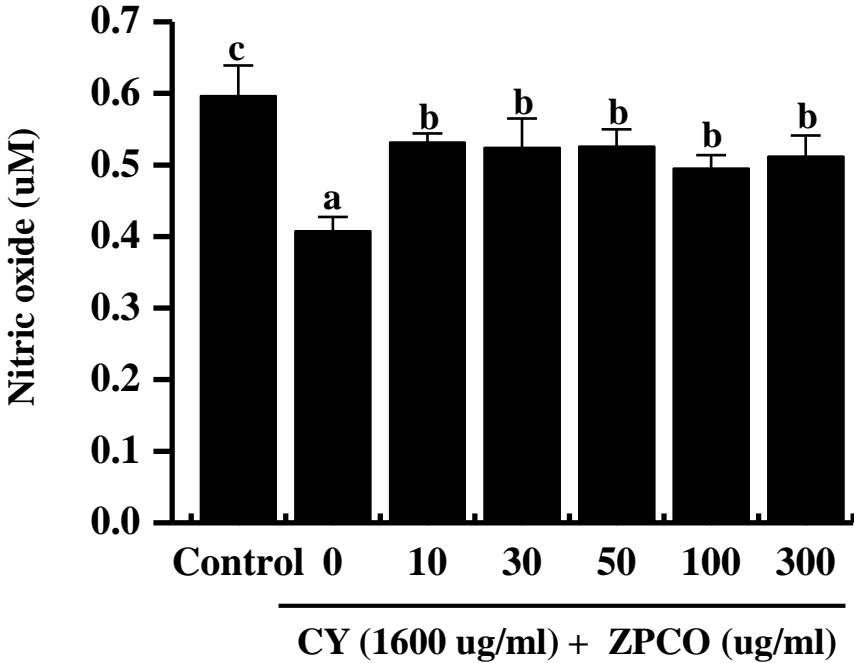

Supplement: Supplementary Materials — Supplementary Figure 1: effect of ZPCO on splenocyte viability. Cells were seeded into 96-well plates, followed by treatment with ZPCO (0, 5, 10, 30, 50, 100, or 300 μg/ml). Next, the cells were incubated for 24 h in a 5% CO2 incubator, after which viability was assessed. Bars labeled with different superscripts have significantly different values (P < 0.05). Data are presented as means ± standard errors (n = 3). Supplementary Figure 2: effect of ZPCO on the concentration of cytokines in splenocytes. Cells were seeded into 96-well plates, followed by treatment with ZPCO (0, 5, 10, 30, 50, 100, or 300 μg/ml). Next, the cells were incubated for 24 h in a 5% CO2 incubator, after which the levels of TNF-α, IFN-γ, IL-2, and IL-12 secretion into the culture medium were analyzed. Bars labeled with different superscripts have significantly different values (P < 0.05 versus control). Data are presented as means ± standard errors (n = 3). Supplementary Figure 3: effects of ZPCO on NO production in splenocytes. Splenocytes were incubated with ZPCO (0, 10, 30, 50, 100, and 300 ug/ml) or cyclophosphamide (1600 μg/ml) for 24 h, and NO concentrations in the culture supernatants were assessed using the Griess assay. Bars labeled with different superscripts have significantly different values (P < 0.05). Data are presented as means ± standard errors (n = 3). [file 8107326.f1.pdf]
